# Adjunctive Catheter-Directed Thrombolysis during Primary PCI for ST-Segment Elevation Myocardial Infarction with High Thrombus Burden

**DOI:** 10.3390/jcm11010262

**Published:** 2022-01-04

**Authors:** Satsuki Noma, Hideki Miyachi, Isamu Fukuizumi, Junya Matsuda, Hideto Sangen, Yoshiaki Kubota, Yoichi Imori, Yoshiyuki Saiki, Yusuke Hosokawa, Shuhei Tara, Yukichi Tokita, Koichi Akutsu, Wataru Shimizu, Takeshi Yamamoto, Hitoshi Takano

**Affiliations:** 1 Division of Cardiovascular Intensive Care, Nippon Medical School Hospital, Tokyo 113-8603, Japan; satsuki-n@nms.ac.jp (S.N.); isamu-f@nms.ac.jp (I.F.); jun1984087@nms.ac.jp (J.M.); sangen777@nms.ac.jp (H.S.); s9012@nms.ac.jp (Y.I.); s8043@nms.ac.jp (Y.S.); y-hosokawa@nms.ac.jp (Y.H.); s5062@nms.ac.jp (S.T.); yukichi@nms.ac.jp (Y.T.); koichi-a@nms.ac.jp (K.A.); wshimizu@nms.ac.jp (W.S.); yamamoto56@nms.ac.jp (T.Y.); 2 Department of Cardiovascular Medicine, Nippon Medical School, Tokyo 113-8603, Japan; ykubota@nms.ac.jp (Y.K.); htakano@nms.ac.jp (H.T.)

**Keywords:** high coronary thrombus burden, tissue plasminogen activator, catheter-directed thrombolysis

## Abstract

Background: High coronary thrombus burden has been associated with unfavorable outcomes in patients with ST-segment elevation myocardial infarction (STEMI), the optimal management of which has not yet been established. Methods: We assessed the adjunctive catheter-directed thrombolysis (CDT) during primary percutaneous coronary intervention (PCI) in patients with STEMI and high thrombus burden. CDT was defined as intracoronary infusion of tissue plasminogen activator (t-PA; monteplase). Results: Among the 1849 consecutive patients with STEMI, 263 had high thrombus burden. Moreover, 41 patients received t-PA (CDT group), whereas 222 did not receive it (non-CDT group). No significant differences in bleeding complications and in-hospital and long-term mortalities were observed (9.8% vs. 7.2%, *p* = 0.53; 7.3% vs. 2.3%, *p* = 0.11; and 12.6% vs. 17.5%, *p* = 0.84, CDT vs. non-CDT). In patients who underwent antecedent aspiration thrombectomy during PCI (75.6% CDT group and 87.4% non-CDT group), thrombolysis in myocardial infarction grade 2 or 3 flow rate after thrombectomy was significantly lower in the CDT group than in the non-CDT group (32.2% vs. 61.0%, *p* < 0.01). However, the final rates improved without significant difference (90.3% vs. 97.4%, *p* = 0.14). Conclusions: Adjunctive CDT appears to be tolerated and feasible for high thrombus burden. Particularly, it may be an option in cases with failed aspiration thrombectomy.

## 1. Introduction

In patients with ST-segment elevation myocardial infarction (STEMI), the preferred reperfusion regimen is primary percutaneous coronary intervention (PCI) [1,2]. However, even in cases where primary PCI is needed, high thrombus burden presents difficulties. High thrombus burden is associated with distal embolization, the slow-/no-reflow phenomenon, abrupt closure, stent thrombosis, and poor prognosis [3,4,5,6]. The therapeutic strategy for STEMI with high thrombus burden includes intracoronary thrombolysis, aspiration thrombectomy, distal embolic protection, excimer laser coronary angioplasty, balloon angioplasty, and stenting. However, interventional cardiologists find it challenging to establish an optimal treatment for high thrombus burden. It has been reported in small case series and studies that intracoronary administration of tissue plasminogen activator (t-PA) reduces coronary thrombus and improves thrombolysis in myocardial infarction (TIMI) flow grade [7,8].

Monteplase (Eisai Co. Ltd, Tokyo, Japan) is a mutant t-PA made by substituting only one amino acid in the epidermal growth factor domain and expressed in baby Syrian hamster kidney cells. It has a half-life of >20 min, which is long compared with the native t-PA half-life of four minutes [9], and it can be administered intravenously by a bolus injection. Kawai et al. reported that a single bolus injection of monteplase produces a higher rate of early recanalization of infarct-related coronary arteries than native t-PA [10]. Moreover, several studies have reported the usefulness of intravenous monteplase before PCI in the treatment of acute myocardial infarction [9,11]. Monteplase is classified as a third-generation thrombolytic drug and an intravenous bolus of 0.22 mg (27.500 IU)/kg of monteplase is equivalent to an intravenous infusion of 100 mg of alteplase and an intravenous bolus of 0.5 mg/kg of tenecteplase [12]. However, t-PA is known to cause paradoxical activation of thrombin, clot formation, and bleeding. Therefore, current guidelines recommend fibrinolytic therapy including t-PA within 12 h of symptom onset if primary PCI cannot be performed within 120 min after the diagnosis of STEMI. A T-time study showed that adjunctive low-dose intracoronary alteplase administered during primary PCI does not reduce microvascular obstruction in patients with STEMI within six hours of symptom onset [13]. However, it is unclear whether catheter-directed thrombolysis (CDT) using intracoronary monteplase during primary PCI constitutes effective treatment for patients with STEMI and high thrombus burden. Thus, the aim of this study was to evaluate the safety and feasibility of adjunctive CDT during primary PCI in patients with STEMI and high thrombus burden and to identify suitable candidates for this therapy.

## 2. Materials and Methods

### 2.1. Study Protocol

Between January 2005 and December 2017, 1849 consecutive patients with STEMI were transferred to Nippon Medical School Hospital, Tokyo, Japan. We found that 263 of these patients had high thrombus burden (i.e., an intracoronary thrombus of length >20 mm). Of the 263 patients, 41 were administered adjunctive CDT with intracoronary infusion of t-PA (monteplase) during primary PCI (CDT group) and 222 were not administered the adjunctive therapy during primary PCI (non-CDT group). The diagnosis of STEMI was based on findings of characteristic symptoms of myocardial ischemia, electrocardiographic change (ST-segment elevation in at least two contiguous leads and new-onset complete left bundle branch block), and subsequent release of biomarkers of myocardial necrosis [2]. Following STEMI diagnosis and provision of informed consent, all patients underwent primary PCI according to guideline-based practices with the early use of concomitant antiplatelet and anticoagulant medications. Antiplatelet therapy consisted of aspirin and a thienopyridine derivative (clopidogrel, prasugrel, or ticlopidine). With regard to anticoagulant therapy, intravenous unfractionated heparin (100 U/kg) was administered before primary PCI.

High thrombus burden was defined as the presence of an intracoronary thrombus of length >20 mm that is visible on angiography. If there was complete obstruction of an infarct-related artery by an angiographic thrombus, high thrombus burden was evaluated after minimal coronary flow was recovered after wire cross, aspiration thrombectomy, or balloon angioplasty. In our criteria, high thrombus burden corresponded to TIMI thrombus grades 4 (i.e., definite thrombus with the largest dimension ≥2 vessel diameters) and 5 (i.e., total occlusion) [14].

**Figure 1:**
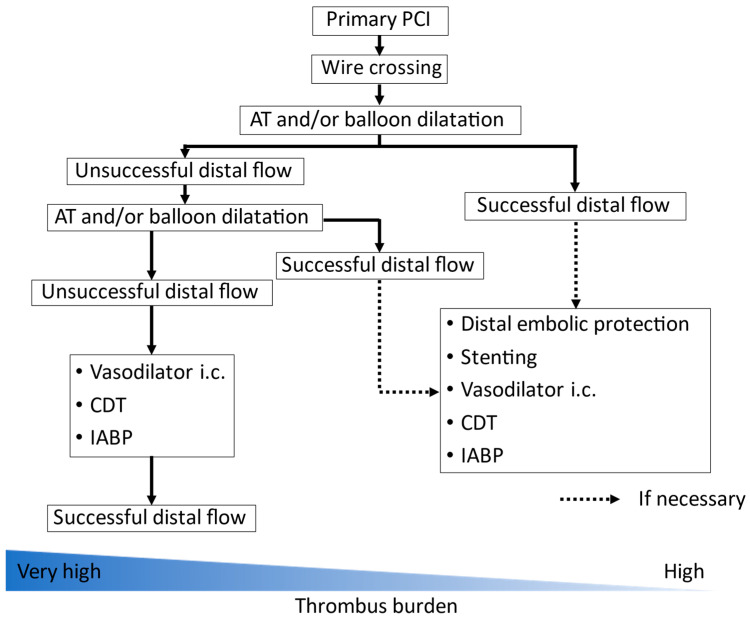
Basic protocol of primary PCI for STEMI patients with high thrombus burden. PCI, percutaneous coronary intervention; AT, aspiration thrombectomoy; CDT, catheter-directed thrombolysis; IABP, intra-aortic balloon pump.

The PCI strategy depended on individual interventional cardiologists, and CDT was performed according to their judgements. However, in our hospital, the basic treatment protocol for STEMI patients with high thrombus burden is defined as follows (Figure 1). The final goal of the primary PCI is coronary reperfusion and not complete removal of the thrombus. First of all, aspiration thrombectomy is often performed for the reduction in thrombus volume in cases where coronary flow can be occluded even after wire crossing, when it is limited to STEMI patients with high thrombus burden. Balloon dilation may be preferred in cases where coronary flow can be resumed just by wire crossing. If successful distal flow is obtained, PCI may be completed without stent implantation. If distal flow is unsuccessful, thrombus aspiration and balloon dilation are repeatedly performed. If necessary, stents may also be implanted with distal embolic protection. If coronary flow is still inadequate even after repeated balloon dilatation and/or aspiration thrombectomy, intracoronary injection of nitroprusside/nicorandil and/or CDT are added, or intra-aortic balloon pump (IABP) is inserted. In this study, we focused the adjunctive CDT during primary PCI.

The following data were collected and compared between the two groups: medical history, coronary risk factors, clinical characteristics, coronary angiographic findings, therapeutic strategies, PCI procedures, bleeding complications, in-hospital mortality, long-term mortality, major adverse cardiac events (MACEs), TIMI flow grades, myocardial blush grade, and corrected TIMI frame count. MACE was defined as all causes of death, reinfarction, and ischemia-driven target vessel revascularization. TIMI flow grade and corrected TIMI frame count (cTFC) were measured as the assessment of coronary flow [15,16]. Myocardial blush grade was measured as the parameter of myocardial reperfusion after PCI [17]. Data on bleeding complication as defined using TIMI bleeding criteria were collected [18]. TIMI major bleeding was defined as bleeding leading to death, intracranial bleeding, or a decrease in hemoglobin level greater than 5 g/dL from the baseline. TIMI minor bleeding was defined as spontaneous and observed blood loss with a decrease in hemoglobin level greater than 3 g/dL, but less than 5 g/dL from baseline or unobserved blood loss with a decrease in hemoglobin level greater than 4 g/dL but less than 5 g/dL from baseline [19]. We assessed TIMI bleeding criteria as the safety and in-hospital and long-term mortalities and long-term MACE as the feasibility of CDT. TIMI flow grade, cTFC, and myocardial blush grade were assessed as the efficacy of additional CDT. 

### 2.2. Statistical Analysis

All continuous variables are presented as means and standard deviations. Categorical variables are presented as numbers or percentages. Categorical variables were tested using the Chi-square test or Fisher’s exact test. Continuous variables were tested using Student’s *t*-test or the Mann–Whitney U test. To evaluate MACE and long-term mortality, we compared the Kaplan–Meier curve using the log-rank test. Propensity score matching analysis was performed to assess the association between t-PA and outcomes to balance for risk factors. A propensity score for t-PA was generated based on a multivariable logistic regression model using the following variables: age, sex, body mass index, Killip class, hypertension, dyslipidemia, diabetes mellitus, and smoking. Propensity score matching was conducted using 4-digit nearest neighbor matching with a 0.20 caliper and a 1:1 match ratio. Values of *p* < 0.05 were considered to be statistically significant. All statistical analyses were performed using IBM SPSS statistics 26 (IBM Corporation, Armonk, NY, USA).

## 3. Results

### 3.1. Study Population

Data on the baseline characteristics of the CDT and non-CDT groups are shown in Table 1. The CDT group had a higher percentage of men and a lower mean age than the non-CDT group. There were no significant differences in blood pressure, heart rate, maximum creatine kinase level, and maximum creatine kinase-MB level between the two groups. The usage rates of aspirin, thienopyridine derivatives, and unfractionated heparin were similar between the two groups. A similarly high proportion of patients in both groups had coronary risk factors.

### 3.2. Angiographic Findings, Procedural Data, Outcomes, and Complications

Data on angiographic findings, PCI technical procedures, and TIMI flow grade before and after PCI are shown in Table 2. About 68% of culprit lesions in the CDT group and 57.2% of the culprit lesions in the non-CDT group were found in the right coronary artery (RCA). With regard to PCI procedures, a significantly higher contrast medium volume was used in the CDT group than in the non-CDT group (219.0 ± 56.9 mL vs. 182.1 ± 64.0 mL, *p* = 0.02). Radiation time was significantly longer in the CDT group than in the non-CDT group (45.0 ± 26.4 min vs. 35.3 ± 21.8 min, *p* = 0.02). As described in the Materials and Methods, PCI was performed according to each interventional cardiologist’s judgement, however, PCI strategy was based on the basic protocol. Aspiration thrombectomy was performed in over 90% of patients in both groups. The majority of patients in both groups underwent balloon dilatation (80.5% vs. 73.9%, *p* = 0.48). There were no significant differences in the use of distal protection devices (19.5% vs. 21.6%, *p* = 0.76). Significantly fewer stents were used in the CDT group than in the non-CDT group (58.5% vs. 96.8%, *p* < 0.01). There were no significant differences on intracoronary administration of nitroprusside or nicorandil (24.4% vs. 15.8%, *p* = 0.18). The use frequency of IABP was significantly higher in the CDT group than in the non-CDT group (41.5% vs. 21.2%, *p* < 0.01). In this manner, patients in the CDT group tended to require more procedures than non-CDT group. However, in those patients, stent implantation was avoided because of the risk of distal embolization and in-stent thrombus protrusion due to residual thrombus.

**Figure 2:**
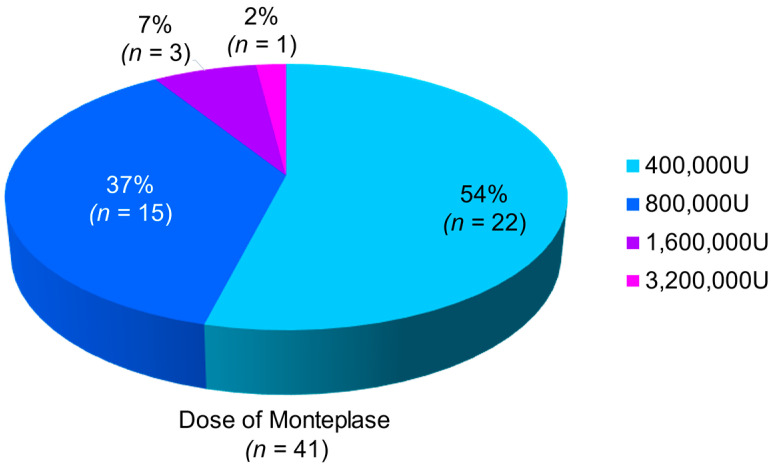
Dose of monteplase administered and proportion of patients.

Regarding the invasive therapeutic strategy, when coronary flow did not improve sufficiently after various invasive procedures, adjunctive CDT was performed. To definitely inject monteplase into the coronary artery, intracoronary monteplase was administered using a guiding catheter (*n* = 24) and other catheters such as microcatheters and aspiration catheters (Lumine infusion catheter: *n* = 11; Thrombuster II or III: *n* = 5; Rebirth: *n* = 2; Eliminate: *n* = 1; Pronto V3: *n* = 1; ST01: *n* = 1) (Table 3). The mean dose was 702,439 ± 519,850 U. Each dose is shown in Figure 2, which was used within the insurance coverage. Monteplase was initially injected as a single bolus (400,000 IU) and added with each additional bolus.

**Figure 3:**
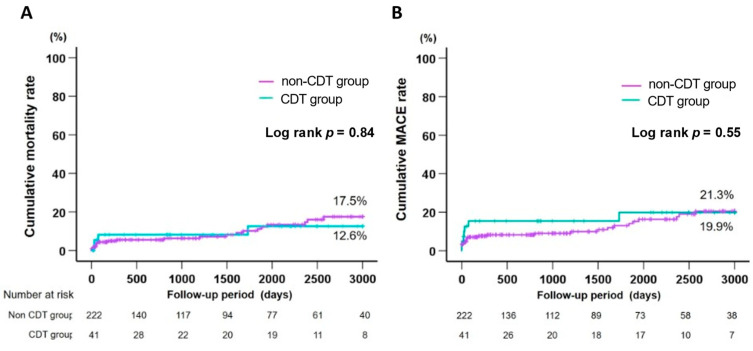
Cumulative mortality rates (**A**) and MACE rates (**B**) in the CDT and non-CDT groups. Kaplan–Meier curves for cumulative mortality rate and MACE (death, reinfarction, or ischemia-driven target vessel revascularization) rate are shown in Figure 3A and Figure 3B, respectively. CDT, catheter-directed thrombolysis; MACE, major adverse cardiac event.

The TIMI major and minor bleeding rates were not significantly different between the two groups (Table 2). In-hospital mortality was also similar between the two groups (7.3% versus 2.3%, *p* = 0.11) (Table 2), and there was no significant difference in the cumulative mortality rate between both groups (12.6% versus 17.5%, log-rank *p* = 0.84) (Figure 3A). Furthermore, there was no significant difference in MACEs between the two groups (19.9% versus 20.5%, log-rank *p* = 0.55) (Figure 3B).

As shown in Table 2, approximately 90% of patients in both groups had TIMI grade 0 flow before PCI. After PCI, the proportion of patients with TIMI grade ≥2 flow increased from 5.0% to 98.2% in the non-CDT group and from 2.4% to 82.9% in the CDT group. Moreover, there was a significant difference in the distribution of TIMI flow grade after PCI between the two groups (*p* < 0.01). Myocardial blush grade distribution also differed significantly between both groups. Final myocardial blush grade 3 accounted for 55.0% and 26.8% in the non-CDT and CDT groups, respectively. The mean value of cTFC before PCI was high and not different significantly between both groups. The mean value of cTFC decreased after PCI, however, it was higher in the CDT group than in the non-CDT group. Despite adjunctive CDT, coronary flow did not recover as well as those in the non-CDT group.

### 3.3. Propensity Score Matching Analysis

**Figure 4:**
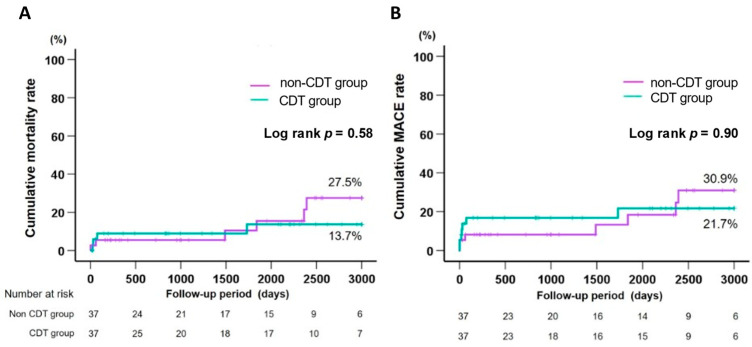
Cumulative mortality rates (**A**) and MACE rates (**B**) after propensity score matching. After propensity score matching, Kaplan–Meier curves for cumulative mortality rate and MACE (death, reinfarction, or ischemia-driven target vessel revascularization) rate are shown in Figure 4A and Figure 4B, respectively. CDT, catheter-directed thrombolysis; MACE, major adverse cardiac event.

Propensity score matching analysis was conducted in selected patients with similar characteristics and comorbidities within each group. After propensity score matching, 74 patients (37 in each group) were included. Baseline patient characteristics and medications of the matched groups are shown in Table 4. Both groups matched well for clinical variables. Even after propensity score matching analysis, in-hospital mortality, major bleeding, and minor bleeding did not differ significantly between both groups (8.1% vs. 5.4%, *p* = 1.00; 5.4% vs. 5.4%, *p* = 1.00; and 10.8% vs. 8.1%, *p* = 1.00, respectively). The Kaplan–Meier curves for cumulative mortality rates and MACEs revealed no differences between the CDT and non-CDT group (13.7% vs. 27.5%, log-rank *p* = 0.58, 21.7% vs. 30.9%, log-rank *p* = 0.90, respectively) (Figure 4).

### 3.4. Antecedent Aspiration Thrombectomy Cases

**Figure 5:**
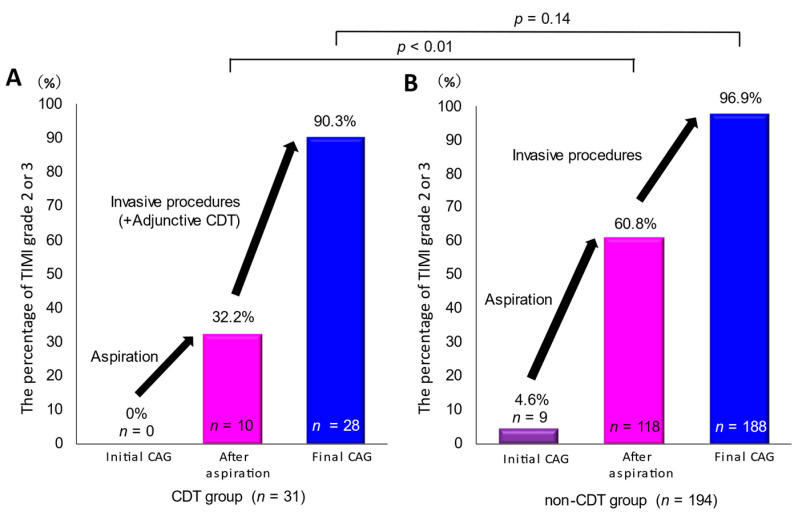
Change of TIMI grade 2 or 3 flow in antecedent aspiration thrombectomy cases. We performed subgroup analysis of antecedent aspiration thrombectomy cases (CDT group: *n* = 31; non-CDT group: *n* = 194). (**A**,**B**) show the rate of TIMI grade 2 or 3 flow during PCI in the CDT group and the non-CDT group, respectively. TIMI, thrombolysis in myocardial infarction; CDT, catheter-directed thrombolysis; IABP, intra-aortic balloon pump.

As described before, aspiration thrombectomy was initially performed to remove high thrombus burden in a number of patients (CDT group: *n* = 31 (75.6%); non-CDT group: *n* = 194 (87.4%)). Subsequently, several therapeutic procedures such as balloon dilatation, distal embolic protection, stenting, intracoronary vasodilator infusion, IABP, and CDT were performed, if necessary. Therefore, we also analyzed antecedent aspiration thrombectomy cases. In most patients in the CDT group, TIMI flow grade did not improve substantially after aspiration thrombectomy. TIMI flow increased to grades 2 or 3 after aspiration thrombectomy in only 32.2% of patients in the CDT group (Figure 5). Thereafter, intracoronary t-PA administration and other procedures were added. As a result, most patients (90.3%) finally had TIMI grade 2 or 3 flow. In contrast, in the non-CDT group, initial aspiration thrombectomy drastically improved TIMI flow grade (TIMI grade 2 or 3 flow: from 4.6% to 60.8%). Finally, TIMI grade 2 or 3 flow was achieved in 96.9% of patients in the non-CDT group, and this was not significantly different compared with that in the CDT group (*p* = 0.14). 

**Figure 6:**
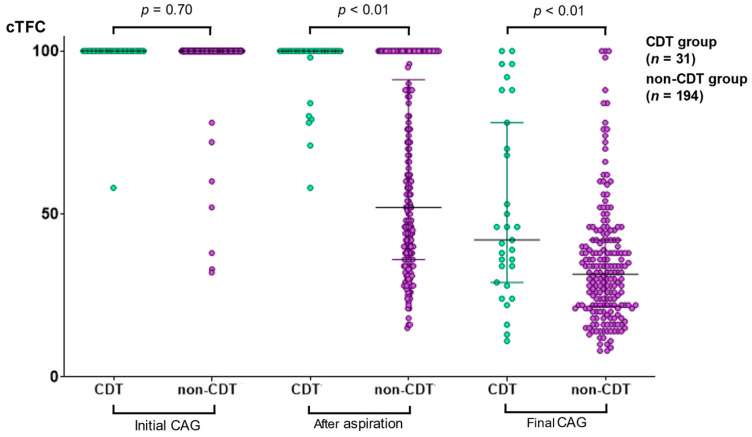
Change in corrected TIMI frame count in antecedent aspiration thrombectomy cases. In antecedent aspiration thrombectomy cases (CDT group: *n* = 31; non-CDT group: *n* = 194), Figure 6 shows the distribution of cTFC at the initial CAG, after aspiration, and final CAG. TIMI, thrombolysis in myocardial infarction; CDT, catheter-directed thrombolysis; CTFC, corrected TIMI frame count; CAG, coronary arteriography; IQR, interquartile range.

On the other hand, cTFC distributions were quite different between both groups (Figure 6). At the initial CAG, cTFCs were 100 in most cases within both groups, and no significant differences between both groups were observed (*p* = 0.70). After aspiration thrombectomy, mean cTFC in the non-CDT group drastically decreased and was significantly lower than that in the CDT group (*p* < 0.01). The final median cTFC in the CDT group also decreased, although it remained higher than that in the non-CDT group (*p* < 0.01). Additionally, the final cTFC in the CDT group was more widely distributed than that in the non-CDT group.

### 3.5. Clinical Characteristics of Patients in the CDT Group with Final TIMI Flow Grade 0 or 1

Despite intracoronary t-PA administration, the final TIMI flow grade of six patients with STEMI and high thrombus burden did not improve but remained at grade 0 or 1. The clinical characteristics of these six patients are shown in Table 5. All patients were men, and half of the infarct-related lesions were distal branch lesions (#4AV lesions in two patients and a #14 lesion in one patient). Four patients had plaque rupture lesions, and two patients had embolism. One patient died due to ventricular septal perforation (patient 5). An RCA #3 lesion with TIMI flow grade 0 was the culprit lesion. Due to the severely tortuous nature of the RCA, devices could not be passed across the lesion. Therefore, the interventional cardiologist administered an intracoronary t-PA injection.

## 4. Discussion

This study revealed several important findings. First, adjunctive CDT during primary PCI for patients with STEMI and high thrombus burden resulted in favorable outcomes. Although the outcomes including in-hospital and long-term mortalities and long-term MACE were not superior to those of patients who did not require adjunctive CDT, it should be considered that adjunctive CDT was performed in refractory patients that coronary flow did not improve even after various invasive procedures. Next, adjunctive CDT improved coronary flow, however, the final coronary flow after adjunctive CDT evaluated by TIMI flow grade and cTFC were inferior to those of patients who did not require adjunctive CDT. Third, limited to patients who underwent antecedent aspiration thrombectomy according to our therapeutic strategy, those in the CDT group had poorer coronary flow after thrombectomy compared to those in the non-CDT group. However, the final coronary flow dramatically improved, and the TIMI flow grade 2 or 3 rate was similar between both groups. This study showed that adjunctive CDT may be an option in selected cases such as patients with failed aspiration thrombectomy.

Historically, urokinase, streptokinase, and t-PA have been used as thrombolysis options. In a previous study in which intracoronary urokinase was administered after thrombus development during PCI, there was no reported in-hospital death, but 10% of the patients required blood transfusions [20]. The results of the Thrombolysis and Angioplasty in Unstable Angina study and those of several other studies show that the effectiveness of intracoronary urokinase in the treatment of stable and unstable anginas is largely discouraging [21,22,23]. In the Intracoronary t-PA Registry, the bleeding complication rate of thrombolysis was shown to be high (9.2%) [24]. As a result, intracoronary thrombolysis is rarely used in clinical practice. Furthermore, there have been advancements in device technologies and pharmacology such as aspiration thrombectomy, distal protection devices, dual antiplatelet therapy, and glycoprotein IIb/IIIa inhibitors [25,26].

Conversely, several other studies support the usefulness of intracoronary thrombolysis in select cases [7,24,27,28]. It was reported in some case reports that adjunctive CDT during PCI is useful as a therapeutic strategy for high thrombus burden [29,30,31,32]. In particular, two case reports showed that post-intracoronary thrombolytic therapy is a good option for patients who previously underwent failed thrombectomy during primary PCI [30,31]. These case findings are consistent with the results of our study. In our study, adjunctive CDT was administered to patients whose coronary flow could not be improved using aspiration thrombectomy. Post-procedurally, the adjunctive CDT strategy led to favorable outcomes, and final coronary flow was almost similar to those of the non-CDT strategy. Therefore, adjunctive CDT may be an option for cases of high thrombus burden in which coronary flow does not improve after aspiration thrombectomy.

Recent studies have revealed the unusefulness of intracoronary thrombolysis in microvascular obstruction due to distal embolization of the thrombus [13,33,34]. Although the current study showed that intracoronary thrombolysis improved the coronary flow, a few cases still had slow flow or no reflow. In such cases, poor coronary flow may be attributed to microvascular obstruction due to distal embolism from proximal high thrombus burden. Moreover, adjunctive CDT has some safety concerns such as bleeding complications. However, previous studies have shown that low-dose intracoronary t-PA administration did not increase the bleeding risk [13,33]. The current study also found no significant difference in bleeding complication between both groups, and mean dose of monteplase in this study corresponded to the low dose in previous studies. However, the rates of bleeding complications were higher in the CDT group than in the non-CDT group, and the number of patients was so small in this study. Thus, further research is required to address concerns over bleeding complications.

The clinical characteristics of patients in the CDT group with final TIMI grade 0 or 1 flow are shown in Table 4. RCA and/or distal lesions accounted for four of six (66%) cases of infarct-related lesions. Thus, even with adjunctive CDT, it may be difficult to obtain good TIMI flow in the case of an RCA lesion with high thrombus burden at the distal branch. To prevent the no-reflow phenomenon, it is important to reduce thrombus burden using thrombectomy devices or other means. However, recent large-scale studies and meta-analyses indicated that routine thrombus aspiration during PCI for STEMI increases the risk of stroke and/or transient ischemic attack and does not improve clinical outcomes [35,36,37,38]. Therefore, routine thrombus aspiration is not recommended in recent guidelines [2]. However, in a previous study, the subgroup analysis of patients with high thrombus burden (i.e., TIMI thrombus grade ≥3) suggested that thrombus aspiration improves cardiovascular mortality [39]. Thus, it seems reasonable that many patients with high thrombus burden initially underwent aspiration thrombectomy in this study. In addition, it was reported in the ASSENT-4 PCI trial that compared with primary PCI, full-dose tenecteplase combined with PCI is associated with an increase in the primary end point of death, congestive heart failure, or shock within 90 days [40]. Thus, adjunctive CDT during PCI should not be routinely recommended but should be limited to patients with STEMI and high thrombus burden, as shown in this study.

Several important limitations of our study should be noted. First, because this study was a retrospective nonrandomized trial with a small sample size that was performed at a single institute, there may be selection bias. In particular, it is noteworthy as a selection bias that additional CDT was performed in selected patients with poor coronary flow after invasive procedures. Additionally, other treatment options such as aspiration thrombectomy, distal embolic protection, IABP, and intracoronary nitroprusside or nicorandil may have contributed to the final patient outcomes. The administration technique for CDT was different and t-PA was performed using a guiding catheter, microcatheter, or aspiration catheter. It was evaluated whether t-PA was effectively delivered to coronary thrombus using any catheter. Second, thrombus burden was higher in this study than in other studies. Previous studies defined high thrombus burden as the presence of accumulated thrombus >3 times the luminal diameter of the infarct-related artery or TIMI thrombus grade >3 [6, 41]. In contrast, we defined high thrombus burden as the presence of an intracoronary thrombus of length >20 mm that is visible in the angiography and corresponds to TIMI thrombus grade >4. This difference may underestimate the effectiveness of adjunctive CDT. In contrast, detailed quantitative evaluation of thrombus burden was not performed in this study. Thus, thrombus burden size may be associated with efficacy of adjunctive CDT strategy. Third, because the efficacy of an intravenous bolus injection of abciximab in the prevention of post-PCI coronary events in Japanese patients has not been confirmed [42], the glycoprotein IIb/IIIa receptor antagonist is not available in Japan and was not used in this study. Additionally, the usage rate of the anticoagulation drug was not analyzed in this study; therefore, its effect may be underestimated. Finally, the pathophysiology of STEMI such as plaque rupture, plaque erosion, or calcified nodules was not considered in this study, which may have had an effect on the results. These limitations deserve confirmation in a large randomized controlled trial.

## 5. Conclusions

Adjunctive CDT during primary PCI is tolerated and feasible for STEMI patients with high thrombus burden. Particularly, it may be a useful therapeutic option in cases of high thrombus burden in which coronary flow cannot be significantly improved using aspiration thrombectomy.

## Figures and Tables

**Table 1:** Patients’ clinical characteristics.

	CDT Group (*n* = 41)	Non-CDT Group (*n* = 222)	*p* Value
Age (years)	59.4 ± 12.8	66.9 ± 13.0	<0.01
Male (%)	92.7	73.0	<0.01
BMI (kg/m^2^)	25.1 ± 4.8	24.2 ± 3.8	0.20
Systolic BP (mmHg)	118.5 ± 32.9	120.3 ± 31.2	0.77
Diastolic BP (mmHg)	68.6 ± 20.4	68.9 ± 19.7	0.94
HR (beats/min)	76.1 ± 23.4	77.4 ± 20.5	0.77
EF (%)	57.6 ± 7.6	49.4 ± 12.6	0.06
**Creatine kinase (CK)**			
Max CK (IU/L)	3467 ± 2999	3314 ± 2649	0.74
Max CKMb (IU/L)	294 ± 308	311 ± 3250.72	
**Cardiovascular history**			
MI (%)	17.1	13.2	0.55
PCI (%)	8.6	14.7	0.42
CABG surgery (%)	2.9	1.5	0.50
Heart failure (%)	2.9	1.5	0.50
Cerebral infarction (%)	11.4	10.3	0.77
Hemodialysis (%)	0.0	1.5	1.00
PAD (%)	2.9	1.5	0.50
**Coronary risk factor**			
Hypertension (%)	58.5	74.8	0.03
Dyslipidemia (%)	53.7	56.8	0.71
Diabetes mellitus (%)	24.4	31.1	0.39
Smoking (%)	63.4	62.2	0.88
Hyperuricemia (%)	19.8	19.5	0.46
**Killip classification**			
Class 1 (%)	70.7	74.3	0.85
Class 2 (%)	14.6	10.8	
Class 3 (%)	4.9	6.8	
Class 4 (%)	9.8	8.1	
**Medication before primary PCI**			
Aspirin (%)	100	97.3	0.59
Thienopyridine (%)	97.6	97.3	1.00
Ticlopidine (%)	36.6	14.4	<0.01
Clopidogrel (%)	48.8	59.5	0.27
Prasugrel (%)	12.2	23.4	0.15
Unfractionated heparin	97.6	100	0.16

CDT, catheter-directed thrombolysis; BMI, body mass index; BP, blood pressure; HR, heart rate; EF, ejection fraction; CK, creatine kinase; MI, myocardial infarction; PCI, percutaneous coronary intervention; CABG, coronary artery bypass grafting; PAD, peripheral artery disease.

**Table 2:** Angiographic findings, PCI procedures, and outcomes.

	CDT Group (*n* = 41)	Non-CDT Group (*n* = 222)	*p* Value
**Culprit lesion**			
RCA (%)	68.3	57.2	0.47
LAD (%)	24.4	35.1	
LCX (%)	7.3	6.3	
LMT (%)	0.0	1.3	
**PCI procedure**			
Onset-to-PCI time (min)	235 (IQR 138–698)	260 (IQR 137–575)	0.90
Devices			
Aspiration thrombectomy (%)	90.2	93.2	0.50
Balloon dilatation (%)	80.5	73.9	0.48
Distal protection (%)	19.5	21.6	0.76
Stent (%)	58.5	96.8	<0.01
Vasodilator i.c. (%)*	24.4	15.8	0.18
IABP (%)	41.5	21.2	<0.01
**Contrast medium**			
Dose (mL)	219.0 ± 56.9	182.1 ± 64.0	0.02
Radiation time (min)	45.0 ± 26.4	35.3 ± 21.8	0.02
**TIMI flow grade before PCI**			
0 (%)	92.7	89.6	0.68
1 (%)	4.9	5.4	
2 (%)	0.0	3.6	
3 (%)	2.4	1.4	
**Final TIMI flow grade**			
0 (%)	9.7	0.0	<0.01
1 (%)	7.3	1.8	
2 (%)	31.7	1.3	
3 (%)	51.2	96.9	
**Final myocardial blush grade**			
0 (%)	19.5	10.4	<0.01
1 (%)	17.1	8.6	
2 (%)	36.6	26.1	
3 (%)	26.8	55.0	
**cTFC before PCI**	96.7 ± 15.0	97.5 ± 11.7	0.74
**cTFC after PCI**	53.7 ± 29.4	33.4 ± 18.2	<0.01
**Outcomes**			
In-hospital mortality (%)	7.3	2.3	0.11
Long-term mortality (%)	12.6	17.5	0.84
**Bleeding complications**			
TIMI major bleeding (%)	4.9	0.9	0.11
TIMI minor bleeding (%)	9.8	7.2	0.53

PCI, percutaneous coronary intervention; IQR, interquartile range; RCA, right coronary artery; LAD, left anterior descending artery; LCX, left circumflex; LMT, left main trunk; IABP, intra-aortic balloon pump; Vasodilator*, Nitroprusside or Nicorandil; i.c., intra-coronary; TIMI, thrombolysis in myocardial infarction.

**Table 3:** t-PA intracoronary administration catheter.

Catheter	*n*
Guiding catheter	24
Lumine infusion catheter^®^, Gadelius Medical K.K., Tokyo, Japan	11
Thrombuster II or III^®^, Kaneka Medix Co., Tokyo, Japan	5
Rebirth^®^, Nipro Co., Osaka, Japan	2
Eliminate^®^, Terumo Co., Tokyo, Japan	1
Pronto V3^®^, Teleflex, Wayne, PA, USA	1
ST01^®^, Terumo Co., Tokyo, Japan	1

t-PA, tissue plasminogen activator.

**Table 4:** Patients’ clinical characteristics and outcomes after propensity score matching.

	CDT Group (*n* = 37)	Non-CDT Group (*n* = 37)	*p* Value
Age (years)	61.0 ± 12.2	63.2 ± 12.0	0.45
Male (%)	94.6	91.9	1.00
BMI (kg/m^2^)	24.6 ± 4.8	24.4 ± 3.7	0.83
Systolic BP (mmHg)	124.6 ± 23.7	127.1 ± 28.0	0.68
Diastolic BP (mmHg)	73.4 ± 15.4	72.4 ± 18.6	0.81
HR (beats/min)	77.8 ± 19.4	81.7 ± 19.6	0.40
EF (%)	51.9 ± 12.2	49.9 ± 13.0	0.52
**Creatine kinase (CK)**			
Max CK (IU/L)	3271 ± 2840	3307 ± 2259	0.95
Max CKMb (IU/L)	295 ± 217	285 ± 181	0.82
**Cardiovascular history**			
MI (%)	13.5	13.5	1.00
PCI (%)	2.7	18.9	0.06
CABG surgery (%)	5.4	0.0	0.49
Heart failure (%)	2.7	5.4	1.00
Cerebral infarction (%)	10.8	10.8	1.00
Hemodialysis (%)	0.0	0.0	
PAD (%)	2.7	0.0	1.00
**Coronary risk factor**			
Hypertension (%)	64.9	67.6	1.00
Dyslipidemia (%)	54.1	45.9	0.64
Diabetes mellitus (%)	27.0	35.1	0.62
Smoking (%)	70.3	64.9	0.80
**Killip classification**			
Class 1 (%)	73.0	70.3	0.49
Class 2 (%)	13.5	5.4	
Class 3 (%)	5.4	18.9	
Class 4 (%)	8.1	5.4	
**Final TIMI flow grade**			
0 (%)	10.8	0.0	<0.01
1 (%)	8.1	5.4	
2 (%)	27.0	13.5	
3 (%)	54.1	81.1	
**Final Myocardial blush grade**			
0 (%)	18.9	24.3	0.08
1 (%)	13.5	8.1	
2 (%)	37.8	16.2	
3 (%)	29.7	51.4	
**cTFC** before PCI	96.3 ± 15.8	96.5 ± 12.7	0.96
**cTFC** after PCI	53.4 ± 30.6	37.3 ± 21.2	0.01
**Outcomes**			
In-hospital mortality (%)	8.1	5.4	1.00
**Bleeding complications**			
TIMI major bleeding (%)	5.4	5.4	1.00
TIMI minor bleeding (%)	10.8	8.1	1.00

CDT, catheter-directed thrombolysis; BMI, body mass index; BP, blood pressure; HR, heart rate; EF, ejection fraction; CK, creatine kinase; MI, myocardial infarction; PCI, percutaneous coronary intervention; CABG, coronary artery bypass grafting; PAD, peripheral artery disease; TIMI, thrombolysis in myocardial infarction; cTFC, corrected TIMI frame count.

**Table 5:** Clinical characteristics in STEMI patients with final TIMI 0 or 1 in the CDT group.

Case	Age (y.o)	Sex	IRL	Prior MI	Killip	Lesion Characteristics	The Reason of t-PA Administration	Other Therapeutic Strategies	Initial TIMI Grade	Death
1	71	M	LAD (#7)	-	3	Embolism	Unsuccessful thrombectomy	Thrombectomy IABP	1	-
2	70	M	RCA (4AV)	-	1	Embolism	Peripheral lesion unsuitable for PCI	-	0	-
3	67	M	LCX (#14)	-	1	Plaque rupture	Acute stent thrombosis	Thrombectomy stenting	0	-
4	58	M	RCA (4AV)	+	2	Plaque rupture	Guide-wire induced coronary dissection	Thrombectomy	1	-
5	70	M	RCA (#3)	-	3	Plaque rupture	Devices were undelivered. (severe tortuous)	IABP	0	+ (VSR)
6	59	M	RCA (#2)	-	1	Plaque rupture	Unsuccessful thrombectomy	Thrombectomy IABP stenting Nitroprusside i.c.	0	-

STEMI, ST-elevation myocardial infarction; t-PA, tissue plasminogen activator; TIMI, thrombolysis in myocardial infarction; IRL, infarct-related lesion; LAD, left anterior descending; RCA, right coronary artery; PCI, percuraneous coronary intervention; LCX, left circumflex; IABP, intra-aortic balloon pump; VSR, Ventricular septal rupture; i.c., Intra-coronary.

## Data Availability

The data presented in this study are available on request from the corresponding author.
